# Treatment outcomes using subcision combined with platelet-rich plasma for atrophic acne scars: a study in Vietnamese patients

**DOI:** 10.1093/skinhd/vzag053

**Published:** 2026-05-26

**Authors:** Lam Van Nguyen, Tan Tan Lam

**Affiliations:** Department of Plastic and Aesthetic Surgery, Faculty of Medicine, Can Tho University of Medicine and Pharmacy, Can Tho City, Vietnam; Centre for Cosmetic Surgery and Skin Care, Can Tho University of Medicine and Pharmacy Hospital, Can Tho City, Vietnam; Faculty of Medicine, Nam Can Tho University, Can Tho City, Vietnam

## Abstract

**Backround:**

Facial atrophic scars significantly impact self-confidence and quality of life. Recently, the combination of ­platelet-rich plasma (PRP) and subcision has been proposed as a potential treatment modality; however, clinical trial results remain inconsistent.

**Objectives:**

To assess the clinical efficacy and safety of combining subcision with PRP therapy for the treatment of facial atrophic acne scars in Vietnamese patients.

**Methods:**

A total of 44 patients with facial atrophic scars of grade 2 or higher were included in the study. Each patient underwent three treatment sessions using a combination of subcision and PRP at 4-week intervals. The patients were evaluated by three independent reviewers through a series of sequential photographs taken 1 month after the third treatment session.

**Results:**

All patients presented with atrophic scars on the cheeks, predominantly mixed scars caused by acne (96%). At evaluation timepoints, the Goodman and Baron grading scale demonstrated significant improvement, with statistically significant differences (*P* < 0.05). Following the treatment regimen of subcision combined with PRP, the majority of patients achieved good-to-excellent responses (96%) and reported satisfaction with the treatment outcomes (96%). Common side effects included pain (93%), mild swelling (41%) and erythema (34%), all of which were transient. The baseline severity of scars (Goodman and Baron) and the presence of rolling scars significantly influenced treatment efficacy (*P* < 0.05).

**Conclusions:**

The combination of subcision and PRP is an effective and safe approach for the treatment of atrophic scars, providing significant improvement and high patient satisfaction.

## Teaser

This study evaluated the efficacy and safety of combining subcision with platelet-rich plasma for treating atrophic acne scars in 44 Vietnamese patients. The combination therapy significantly improved scar grades and patient satisfaction, with only mild and transient side effects. Baseline scar severity and rolling morphology were predictors of better treatment outcomes.

## Graphical Abstract

**Graphical Abstract:**
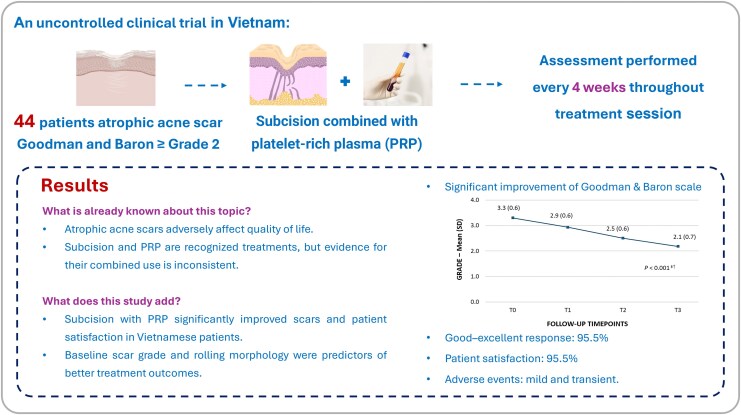

What is already known about this topic?

- Atrophic acne scars adversely affect people’s quality of life.
- Subcision and platelet-rich plasma (PRP) are recognized treatments, but evidence of their combined use is inconsistent.

What does this study add?

- Subcision with PRP significantly improved scars and satisfaction in Vietnamese patients.
- Baseline scar grade and rolling morphology were predictors of better treatment outcomes.

Scars are characterized by a depressed area of the skin accompanied by alterations in structure, pigmentation, vascularization, innervation and chemical properties.^1^ Scar formation can result in hypertrophic or keloid scars due to excessive fibrous tissue formation; however, atrophic scars caused by tissue loss at the injury site are more common.^2,3^ Atrophic scars can arise from various causes, with acne (a chronic inflammatory skin condition affecting millions of people) being the most prevalent.^4^ Facial atrophic scars can profoundly impact an individual’s self-confidence and quality of life, posing significant cosmetic and psychological challenges and leading to anxiety and even depression.^5^

In recent years, platelet-rich plasma (PRP) has emerged as a promising therapeutic modality in dermatology and tissue engineering due to its rich content of growth factors that support dermal regeneration and fibroblast proliferation, which are essential for the management of atrophic scars and chronic wounds.^6–11^ The principle of PRP application in dermatology relies on the regenerative properties of platelets to enhance wound healing and reduce the appearance of acne scars. PRP is a plasma concentrate enriched with platelets and contains numerous growth factors, including platelet-derived growth factor, transforming growth factor, vascular endothelial growth factor and insulin-like growth factor. These growth factors have been shown to stimulate collagen production, promote angiogenesis and modulate inflammation, all of which are essential for tissue regeneration and scar remodelling.^12–14^

Subcision is a minimally invasive procedure that has been demonstrated to improve all three types of acne-related atrophic scars effectively. This technique works by mechanically breaking the fibrous strands tethering the dermis to the underlying subcutaneous tissue, which is responsible for pulling the skin surface downward.^15^ PRP enhances the expression of growth factors, promoting more robust tissue restructuring and creating a synergistic effect when combined with subcision in scar treatment.^16^ In recent years, several controlled clinical trials have been conducted to evaluate the efficacy of combining PRP with subcision to treat atrophic scars. However, the results of these studies remain inconsistent, leaving the actual effectiveness of this combination unclear.^1,7,17^ Therefore, we conducted this study to evaluate the outcomes of treating atrophic scars using subcision combined with PRP. This research aims to clarify the effectiveness of this combination therapy and to provide additional scientific evidence to support the optimization of atrophic scar treatment strategies in clinical practice.

## Patients and methods

### Study settings and participants

This study was an uncontrolled clinical trial conducted in 2023 at the Can Tho University of Medicine and Pharmacy Hospital and Can Tho Dermatology Hospital, major provincial hospitals in the Mekong Delta region in southwestern Vietnam. The primary endpoint was the degree of clinical improvement in facial atrophic acne scars, as assessed following combined subcision and autologous PRP therapy.

Eligible participants included male and female patients aged ≥ 15 years with atrophic facial scars classified as grade ≥ 2, according to the Goodman and Baron grading system.^18^ Only patients who completed the full treatment protocol were included in the final analysis. The study population consisted of individuals presenting with ice pick, rolling and boxcar scars symmetrically distributed on both sides of the face. Patients with a history of atrophic scar treatment using dermal fillers within the past 12 months were excluded to minimize the confounding effects of prior interventions on outcomes. Individuals with a history of keloid formation were also excluded due to the risk of abnormal fibrotic responses that could interfere with scar remodelling. Patients receiving anticoagulant therapy (e.g. warfarin and heparin), those with platelet dysfunction or thrombocytopenia, or with a history of visceral bleeding were excluded not only due to the increased risk of bleeding during subcision, but also because the PRP efficacy is highly dependent on the quantity and functional quality of autologous platelets. Additionally, patients with active facial skin infections, cutaneous malignancies, viral lesions such as warts or premalignant conditions such as actinic keratosis were excluded to prevent local complications and to ensure homogeneity in tissue regeneration responses following treatment.

The required sample size was calculated using the formula $n\;\text{=}\;{\text{Z}}_{\text{1}-\frac{\alpha }{2}}^{\text{2}}\frac{p\;\text{(1}-\;p\text{)}}{d^{\text{2}}}$ where *p* represents the estimated proportion of patients demonstrating a good or better treatment response, *α* is the type I error rate (significance level) and *d* is the desired absolute precision (margin of error). The *P-*value was derived from a prior study by Yadav *et al*.^19^ Assuming a 5% margin of error and a *Z*-value of 1.96 (95% confidence), the minimum sample size was determined to be 31 participants. Ultimately, 49 patients entered the study; 5 did not complete the protocol, leaving 44 patients in the final analysis.

### Patient evaluation

At baseline (T0), all patients underwent a medical history review and dermatological examination to rule out active infections or other dermatological conditions. Skin type, aetiology, lesion location, morphology, pigmentation, scar type and scar severity were assessed using the Goodman and Baron grading scale: grade 1 includes macular erythematous scars with hypo- or hyperpigmentation; grade 2 refers to mild atrophy that is not apparent at social distances >50 cm or is easily covered by facial makeup or beard hair; grade 3 denotes moderate atrophy evident at social distances >50 cm, not easily covered by makeup or beard hair but able to be flattened by manual stretching; and grade 4 indicates severe atrophy that cannot be flattened by manual stretching of the skin.^18^ Scar severity was subsequently evaluated at 4 weeks after the first treatment session (T1), 4 weeks after the second treatment session (T2) and 4 weeks after the final treatment session (T3). At T3, treatment outcomes were assessed based on the degree of improvement, treatment response and scar severity.

Improvement was assessed by three independent dermatologists using photographs taken at different timepoints (T0 and T3). Digital images were captured with a mobile camera (48 MP) at full high-definition resolution, positioned at a 90-degree angle from a distance of 50 cm under daylight standardized conditions. Evaluators used a physician quartile grading scale as follows: mild improvement (0–25%), moderate improvement (26–50%), marked improvement (51–75%) and excellent improvement (76–100%).^7^

Furthermore, an objective assessment of treatment response was conducted based on Goodman and Baron’s qualitative acne scar grading scale.^18^ An improvement of two or more grades or a return to grade 1 was categorized as excellent, an improvement of one grade was categorized as good and no change was categorized as poor.^20^ The Goodman and Baron grading scale, as assessed independently by the three dermatologists at each timepoint (T0–T3), demonstrated a high level of interrater agreement. Specifically, we calculated pairwise weighted kappa coefficients for each combination of evaluators, whom we anonymously designated as Evaluator A, Evaluator B and Evaluator C. The comparisons included A vs. B, A vs. C and B vs. C, analysed with SPSS version 27 (IBM, Armonk, NY, USA). This approach was selected because it is appropriate for ordinal scales and compatible with the statistical platform used in this study. The weighted kappa values for the three evaluator pairs ranged from 0.74 to 0.81, indicating substantial to almost-perfect agreement based on Landis and Koch’s interpretative criteria.^21^

Each participant was also asked to rate their overall satisfaction with the treatment for each side of the face after each session and at the end of the follow-up period using a visual analogue scale. Patient satisfaction was classified as follows: unsatisfied (0%), slightly satisfied (<25%), satisfied (25–60%) and very satisfied (>60%).^7^

### Treatment protocol

#### Procedure of subcision

The skin was cleansed with alcohol, and a surgical pen marker was used to outline the scars to be treated. Following the injection of lidocaine 2% intralesionally, an 18-G needle attached to a 5-mL syringe was inserted at a shallow angle with the bevel facing upward at the periphery of the scarred area. The free hand was used to stabilize the treatment site while the needle was moved in a back-and-forth motion. The needle was then manoeuvred laterally in a fanning motion until no resistance was felt, indicating the endpoint. Firm pressure was applied for at least 5 min to achieve haemostasis.^7^

#### Platelet-rich plasma procedure

To obtain PRP, 20 mL venous blood was collected from the antecubital vein under complete aseptic conditions. The whole-blood sample was drawn into tubes containing sodium citrate (10:1) as an anticoagulant. The citrated blood was then subjected to a two-step centrifugation process. The first centrifugation was performed at approximately 161 × ***g*** for 15 min, separating the blood into three layers: plasma supernatant, buffy coat and red blood cells. The plasma supernatant was then subjected to a second centrifugation at approximately 447 × ***g*** for 10 min, resulting in the separation of plasma into two components: platelet-poor plasma and PRP, with the PRP being the lower 1–2 mL of the plasma layer. The PRP was activated by adding calcium chloride in a 1:9 ratio (0.1 mL CaCl_2_ per 1 mL PRP) immediately before injection. PRP was injected intradermally into each scar at 0.1–0.2 mL per site using an insulin syringe.^19^

### Statistical analyses

The collected data were statistically analysed using SPSS version 27 (IBM). Categorical variables were summarized as frequencies and percentages. Continuous variables were assessed for normality using the Kolmogorov–Smirnov and Shapiro–Wilk tests, which determined the appropriate statistical approach. Normally distributed data (age) were reported as mean (SD), while ­non-normally distributed variables (scar duration) were presented as median and interquartile range. The Freeman–Halton exact test was applied for comparisons of sex, Fitzpatrick skin type, Goodman and Baron grading,^18^ and scar morphology among groups. The Kruskal–Wallis test was used to analyse differences in scar duration across the three response groups (poor, good and excellent). Changes in Goodman and Baron scale scores over time were evaluated using the paired samples *t*-test. A *P*-value <0.05 was considered statistically significant.

## Results

A total of 44 patients were enrolled and followed until the completion of the study, with a nearly equal sex distribution (52% female and 48% male) and a mean (SD) age of 28.3 (1.52) years. Other characteristics are detailed in Table 1, with the majority of patients having Fitzpatrick skin type III and the primary cause of atrophic scars being acne. The mean (SD) age of acne onset was 20.0 (3.8) years and the median scar duration was 7.5 years. Table 2 indicates universal cheek involvement, frequent temple and forehead involvement, and a predominance of mixed morphology, with boxcar scars more common than rolling and ice pick.

**Table 1:** Patient characteristics

Characteristic		*n* = 44
Sex	Male	21 (48)
	Female	23 (52)
Fitzpatrick skin type	Type III	29 (66)
	Type IV	15 (34)
Causes of atrophic scars	Postacne scar	42 (95)
	Traumatic scar	1 (2)
	Varicella scar	1 (2)
Age of onset (years), mean (SD)		20.0 (3.8)
Age of onset (years), range		14–29
Scar duration (years), median (IQR)		7.5 (3–11)

Data are provided as *n* (%) unless otherwise stated. IQR, interquartile range.

**Table 2:** Characteristics of atrophic scars

Characteristic		*n* (%)
Location	Cheek	44 (100)
	Forehead	23 (52)
	Chin/jaw	16 (36)
	Nose	18 (41)
	Temple	33 (75)
Morphological characteristics	Ice pick	23 (52)
	Rolling	34 (77)
	Boxcar	41 (93)
	Mixed	39 (89)
Colour	Normal	29 (66)
	Scar erythema	10 (23)
	Hyperpigmentation	5 (11)

**Figure 1:**
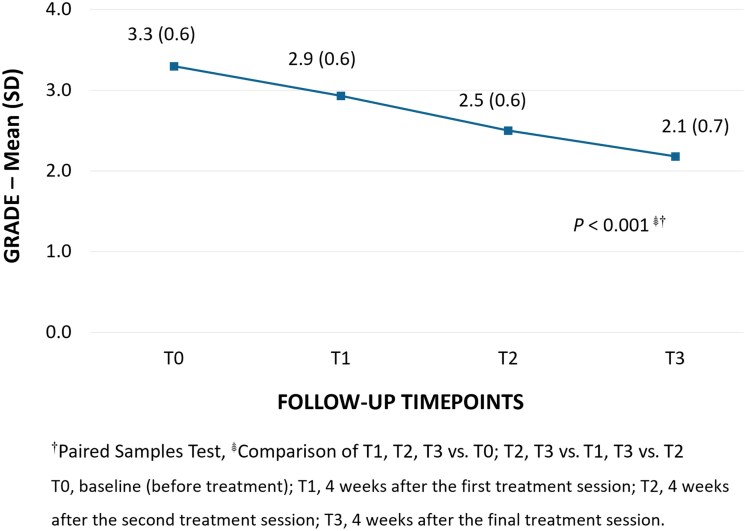
Changes in mean Goodman and Baron acne scar grade across follow-up timepoints.^18^

Scar severity on the Goodman and Baron scale declined progressively across visits (Figure 1),^18^ with a significant overall improvement from baseline to the final assessment (*P* < 0.001). Consistent with this, Table 3 shows that physician-rated improvement was mainly marked or moderate, and patient response was largely good or excellent. Adverse events were as expected, mild and self-limited, principally pain, oedema and erythema, with rare hyperpigmentation; most patients reported being satisfied after treatment.

**Table 3:** Treatment results and side effects

Variable	*n* (%)
Improvement (based on physician’s quartile grading scale)	
Mild	5 (11)
Moderate	11 (25)
Marked	28 (64)
Excellent	0 (0)
Patient’s response (based on Goodman and Baron grade change)^18^	
Poor	2 (5)
Good	32 (73)
Excellent	10 (23)
Side effects	
Pain^a^	41 (93)
Oedema^a^	18 (41)
Erythema^a^	15 (34)
Hyperpigmentation	1 (2)
Patients satisfaction	
Unsatisfied	2 (5)
Slightly satisfied	10 (23)
Satisfied	26 (59)
Very satisfied	6 (14)

^a^All symptoms were self-limited and subsided within 48 h post-treatment.

As summarized in Table 4, a higher baseline Goodman and Baron grade and the presence of rolling scars were associated with better patient-rated response, whereas other morphologies showed no clear signal.

**Table 4:** Clinical factors affecting patient response

Factor			Patient response, *n* (%)			*P*-value
			Poor	Good	Excellent	
Sex		Male	1 (5)	16 (76)	4 (19)	0.86^a^
		Female	1 (4)	16 (70)	6 (26)	
Skin type		Type III	1 (3)	19 (66)	9 (31)	0.19^a^
		Type IV	1 (7)	13 (87)	1 (7)	
Goodman and Baron grade^18^		Grade 2	0 (0)	0 (0)	3 (100)	0.02^a^
		Grade 3	1 (4)	21 (84)	3 (12)	
		Grade 4	1 (6)	11 (69)	4 (25)	
Morphology	Rolling	Yes	0 (0)	24 (71)	10 (29)	0.01^a^
		No	2 (20)	8 (80)	0 (0)	
	Ice pick	Yes	2 (9)	17 (74)	4 (17)	0.49^a^
		No	0 (0)	15 (71)	6 (29)	
	Boxcar	Yes	2 (5)	29 (71)	10 (24)	>0.99^a^
		No	0 (0)	3 (100)	0 (0)	
Scar duration (years), median (IQR)			12.0	8.0 (8.0)	6.5 (9.0)	0.77^b^

^a^Freeman–Halton exact test.

^b^Kruskal–Wallis test. IQR, interquartile range.

**Figure 2:**
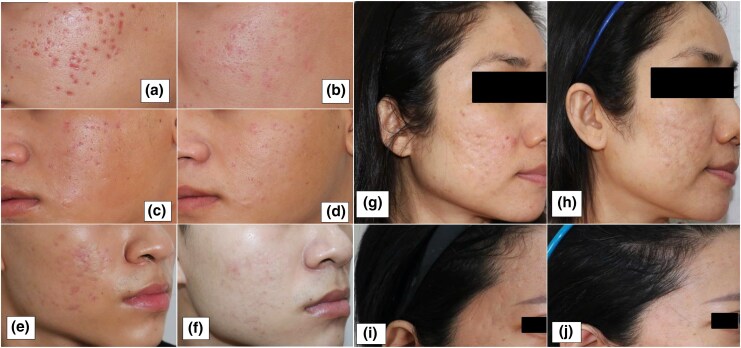
Representative photographs showing clinical improvement in acne scars before and after treatment. (a, c, e, g, i) Baseline photographs of two female patients; (b, d, f, h, j) post-treatment photographs in the same two patients after three sessions.

Figure 2 presents five representative patients who underwent three treatment sessions using subcision combined with PRP. All patients had bilateral atrophic acne scars, predominantly mixed types with a combination of boxcar, rolling and ice pick morphology with Goodman and Baron grades improving by 1–2 levels and consistently high patient satisfaction. The clinical details for each case are as follows.

Patient 1 was a 25-year-old man with predominantly boxcar and rolling scars of Goodman and Baron grade 3 at baseline. After three treatment sessions, the scars improved to grade 1, with visible flattening and textural improvement. The patient reported being very satisfied with the outcome.

Patient 2 was a 20-year-old man with rolling scars of baseline grade 3 severity. Post-treatment, the scars improved to grade 2. The improvement was moderate, and the patient reported being satisfied.

Patient 3 was a 23-year-old man with mixed scars (boxcar and ice pick), initially graded 4 on the Goodman and Baron scale. The scars improved to grade 2, with marked improvement in depth and appearance. The patient reported being very satisfied.

Patient 4 was a 37-year-old woman with deep boxcar scars of grade 4 severity. After three sessions, the scars improved to grade 2, with noticeable contour softening and smoother. The patient reported being satisfied with the outcome.

Patient 5 was a 25-year-old woman with mild rolling scars of grade 2 severity at baseline. Post-treatment, the scars improved to grade 1, and the patient was very satisfied.

## Discussion

During the study, 44 patients with atrophic scars of ≥ grade 2, predominantly mixed scars on the cheeks caused by acne, were treated with subcision and PRP. The outcomes showed that patients experienced an improvement in Goodman and Baron grades and favourable response rates, and were satisfied with the results. Common side effects included transient pain, erythema and oedema, all of which resolved spontaneously.

Atrophic scars are the most common type of facial scars.^22^ In this study, acne was leading cause of atrophic scars. This finding aligns with most previous studies, although postvaricella infections, trauma, burns or postsurgical procedures can also result in atrophic scars.^23,24^ Additionally, the cheeks, temples and forehead were most common sites for atrophic scars, consistent with the retrospective analysis by Kim *et al*. on facial scar morphology.^22^ Furthermore, boxcar scars were the most prevalent, while rolling and ice pick scars were less common. However, most patients had more than one type of scar. These results differ from some previous studies. For example, Yadav *et al*. reported observing only boxcar and rolling scars in patients with acne scars, while the study by Ibrahim *et al.* noted that mixed scars (boxcar and ice pick) accounted for 18.5%, higher than boxcar scars alone (14.8%) but lower than ice pick (37%) and rolling scars (29.6%).^19,23^ Nevertheless, some studies, such as those by Saleh and Alkhayer and Li *et al.*, reported findings consistent with ours.^25,26^ In addition, scars showed normal pigmentation, consistent with the findings of Kim *et al.* on facial scar colouration.^22^

Various treatments for scar management have been reported, with variable outcomes. Among these, subcision is an effective technique for atrophic scars, while autologous PRP has been proposed as a promising adjunct for acne scars.^20,27^ In our study, combined subcision and PRP produced significant, safe improvements and high patient satisfaction. Notably, the Goodman and Baron grades improved significantly over time (*P* < 0.05).^18^ The majority of participants demonstrated marked or moderate improvement according to the physician’s quartile grading scale, while patient-reported outcomes were rated as good or excellent. These findings align with previous studies.^19,20,23,28,29^ For example, a randomized controlled trial (RCT) comparing subcision combined with autologous PRP to subcision alone showed that the combination group achieved a fourfold more significant improvement in Goodman and Baron grade compared with the control group (*P* = 0.004). Subjective patient evaluations also improved by 45.28% in the combination group vs. 20.74% in the control group (*P* = 0.03).^17^ Similarly, another study comparing subcision combined with microneedling and PRP to subcision, ­microneedling and PRP alone reported significantly better results with the PRP combination (*P* = 0.03).^1^ Additionally, a meta-analysis of 311 patients demonstrated that subcision combined with PRP achieved superior clinical outcomes compared with subcision alone or subcision with microneedling.^6^ The efficacy of PRP is attributed to growth factors, cytokines and chemokines released from the alpha granules in concentrated platelets, which stimulate the regeneration of collagen and elastin fibres. The improvement in skin appearance is further facilitated by the stimulation of adipose-derived stem cells, dermal fibroblasts, type I collagen and basement membrane proteins.^12^ Subcision works by releasing fibrous bands at the dermis–subcutaneous interface, lifting the dermis and stimulating wound healing, which promotes connective tissue growth to fill the scarred area. This technique also homogenizes fat lobules and redistributes subcutaneous tension, minimizing contour deformities caused by subcutaneous fat loss.^20^ The combination of PRP with subcision has demonstrated synergistic effects, providing comprehensive improvements in the appearance of atrophic scars and proving a reliable treatment option in clinical practice.

Since PRP therapy utilizes the patient’s own autologous blood components and subcision is a minimally invasive technique, the risk of allergic reactions or side effects is typically minimized.^30,31^ However, as with any medical procedure, there remains the potential for side effects and complications. Consistent with our findings, many studies have reported common side effects at the injection or treatment site, including transient erythema, swelling, bruising and mild pain typically resolving within 2–3 days.^1,6,17^ These symptoms usually resolve spontaneously within a few days. Serious complications such as infections, allergic reactions, or vascular and nerve damage are exceedingly rare. Nonetheless, these risks can be significantly reduced by adhering strictly to aseptic protocols, using high-quality equipment, and ensuring the procedure is performed by experienced physicians.^17,32^

Baseline characteristics may influence treatment efficacy.^33^ Our study found that baseline Goodman and Baron scar grades and scar morphology were associated with response. Grade 2 and rolling scars exhibited lower poor-response rates. This probably reflects the milder severity of grade 2 scars, making patients more likely to respond positively after completing the treatment regimen. Moreover, subcision is the preferred method for rolling scars. Adding PRP may yield better responses than in ice pick or boxcar scars.^34^ Mechanistically, subcision releases tethering bands, elevates the scar and promotes neocollagenesis. Observations by Deshmukh and Belgaumkar also demonstrated superior responses in rolling scar patients treated with subcision and PRP (*P* = 0.001).^17^ Therefore, scar subtype and baseline grade should guide treatment selection.

The strengths of this study include multicentre design, standardized Goodman and Baron assessment, and a standardized protocol with mild, transient adverse effects. Despite these significant findings, the study has some limitations. The sample size was relatively small (44 patients), which may limit the generalizability of the results. Additionally, the follow-up period was restricted to 3 months after the final treatment session, insufficient to assess long-term efficacy or the potential recurrence of scars. Lastly, the study did not include a control group to compare the effectiveness of different treatment modalities, limiting the ability to determine the specific role of PRP within the treatment protocol. The principal limitation is the single-arm design, lacking a parallel control group (e.g. subcision alone), which constrains causal inference about any incremental benefit attributable to adding PRP to subcision. Future adequately powered multicentre RCTs with longer follow-up, standardized PRP parameters and blinded outcomes (including validated patient-reported outcome measures and optional three-dimensional imaging) are warranted.

The combination of subcision and PRP is an effective therapy for treating atrophic scars on the cheeks caused by acne, showing significant improvement according to the Goodman and Baron grading scale and high patient satisfaction. Common side effects such as pain, erythema and mild swelling were transient and had no long-term impact. Additionally, the results indicated that patients with grade 2 scars and rolling scars achieved the best treatment outcomes.

## References

[1] Vashisht A, Krishna A, Chugh R et al PRP and its benefit as an adjunctive therapy with subcision and microneedling in atrophic scars: a comparative study. J Cutan Aesthet Surg 2024; 17:137–45. 38800810 10.4103/JCAS.JCAS_64_23 PMC11126227

[2] Connolly D, Vu HL, Mariwalla K, Saedi N. Acne scarring – pathogenesis, evaluation, and treatment options. J Clin Aesthet Dermatol 2017; 10:12–23. PMC5749614 29344322

[3] Van Nguyen L, Ly HQ, Vo HT et al Clinical features and the outcome evaluations of keloid and hypertrophic scar treatment with triamcinolone injection in Mekong Delta, Vietnam – a cross-sectional study. Clin Cosmet Investig Dermatol 2023; 16:3341–8. 10.2147/CCID.S432735 PMC10661894 38021426

[4] García PN, Andrino RL. Resurfacing of atrophic facial acne scars with a multimodality CO_2_ and 1570 nm laser system. J Cosmet Dermatol 2024; 23:13–18. 38587296 10.1111/jocd.16283

[5] Tan J, Beissert S, Cook-Bolden F et al Impact of facial atrophic acne scars on quality of life: a multi-country population-based survey. Am J Clin Dermatol 2022; 23:115–23. 34705166 10.1007/s40257-021-00628-1 PMC8776674

[6] Long T, Gupta A, Ma S, Hsu S. Platelet-rich plasma in noninvasive procedures for atrophic acne scars: a systematic review and meta-analysis. J Cosmet Dermatol 2020; 19:836–44. 32061047 10.1111/jocd.13331

[7] Hassan AS, El-Hawary MS, Abdel Raheem HM et al Treatment of atrophic acne scars using autologous platelet-rich plasma vs combined subcision and autologous platelet-rich plasma: a split-face comparative study. J Cosmet Dermatol 2020; 19:456–61. 31241854 10.1111/jocd.13048

[8] White C, Brahs A, Dorton D, Witfill K. Platelet-rich plasma: a comprehensive review of emerging applications in medical and aesthetic dermatology. J Clin Aesthet Dermatol 2021; 14:44–57. PMC8675348 34980960

[9] Moftah NH, Mansour AM, Ibrahim SMA. Clinical evaluation of efficacy of intralesional platelet-rich plasma injection versus 1064 nm long-pulsed Neodymium:YAG laser in the treatment of inflammatory acne vulgaris in adolescent and post-adolescent patients: a prospective randomized split-face comparative study. Lasers Med Sci 2022; 37:2471–8. 35084634 10.1007/s10103-022-03510-6 PMC9232433

[10] Conese M, Annacontini L, Carbone A et al The role of adipose-derived stem cells, dermal regenerative templates, and platelet-rich plasma in tissue engineering-based treatments of chronic skin wounds. Stem Cells Int 2020; 2020:7056261. 32399048 10.1155/2020/7056261 PMC7199611

[11] Scarano A, Ceccarelli M, Marchetti M et al Soft tissue augmentation with autologous platelet gel and β-TCP: a histologic and histometric study in mice. Biomed Res Int 2016; 2016:2078104. 27478828 10.1155/2016/2078104 PMC4960330

[12] Xu J, Gou L, Zhang P et al Platelet-rich plasma and regenerative dentistry. Aust Dent J 2020; 65:131–42. 32145082 10.1111/adj.12754 PMC7384010

[13] Woo P . Platelet-rich plasma in treatment of scar, atrophy, and sulcus: short- and long-term results. Laryngoscope Investig Otolaryngol 2023; 8:1304–11. 10.1002/lio2.1143 PMC10601577 37899871

[14] Sabry H, Mostafa S, Abdalla S. Platelet-rich plasma in acne scar: a comprehensive review. Benha J Appl Sci 2023; 8:147–54.

[15] Vempati A, Zhou C, Tam C et al Subcision for atrophic acne scarring: a comprehensive review of surgical instruments and combinatorial treatments. Clin Cosmet Investig Dermatol 2023; 16:125–34. 10.2147/CCID.S397888 PMC9868281 36698445

[16] Schoenberg E, O’Connor M, Wang JV et al Microneedling and PRP for acne scars: a new tool in our arsenal. J Cosmet Dermatol 2020; 19:112–14. 31070298 10.1111/jocd.12988

[17] Deshmukh NS, Belgaumkar VA. Platelet-rich plasma augments subcision in atrophic acne scars: a split-face comparative study. Dermatol Surg 2019; 45:90–8. 30102625 10.1097/DSS.0000000000001614

[18] Goodman GJ, Baron JA. Postacne scarring: a qualitative global scarring grading system. Dermatol Surg 2006; 32:1458–66. 17199653 10.1111/j.1524-4725.2006.32354.x

[19] Yadav MK, Soni P, Ghiya BC et al Efficacy of autologous platelet rich plasma with subcision vs platelet rich plasma with microneedling in atrophic acne scars: a single-center, prospective, intra-individual split-face comparative study. J Cutan Aesthet Surg 2024; 17:124–30. 38800819 10.4103/JCAS.JCAS_218_22 PMC11126223

[20] Minh PPT, Bich DD, Hai VNT et al Microneedling therapy for atrophic acne scar: effectiveness and safety in Vietnamese patients. Open Access Maced J Med Sci 2019; 7:293–7. 30745984 10.3889/oamjms.2019.098 PMC6364723

[21] Landis JR, Koch GG. The measurement of observer agreement for categorical data. Biometrics 1977; 33:159–74. 843571

[22] Kim GH, Lee WJ, Jung JM et al Morphological characteristics of facial scars: a retrospective analysis according to scar location, onset, age, and cause. Int Wound J 2024; 21:e14453. 38058010 10.1111/iwj.14453 PMC10958093

[23] Ibrahim ZA, El-Ashmawy AA, Shora OA. Therapeutic effect of microneedling and autologous platelet-rich plasma in the treatment of atrophic scars: a randomized study. J Cosmet Dermatol 2017; 16:388–99. 28504480 10.1111/jocd.12356

[24] Jin W, Li Z, Jin Z, Jin C. A novel technique for treating atrophic facial scars in Asians using ultra-pulse CO_2_ laser. J Cosmet Dermatol 2020; 19:1099–104. 32073746 10.1111/jocd.13335

[25] Saleh LH, Alkhayer AE. Skin microneedling alone versus skin microneedling plus platelet rich plasma PRP in the treatment of atrophic post acne scars (a split face comparative study). J Medical Pharmaceutical Sci 2022; 6:68–75.

[26] Li J, Duan F, Kuang J et al Clinical factors influencing the effectiveness of microplasma fractional radiofrequency treatment for atrophic acne scars: a retrospective analysis. J Cosmet Dermatol 2024; 23:2433–42. 38532647 10.1111/jocd.16298

[27] Cruciani M, Masiello F, Pati I et al Platelet rich plasma use for treatment of acne scars: an overview of systematic reviews. Blood Transfus 2024; 22:226–38. 37677095 10.2450/BloodTransfus.536 PMC11073618

[28] Nassar A, El-Shaarawy W, Salah E. Autologous plasma gel injection combined with scar subcision is a successful technique for atrophic post-acne scars: a split-face study. J Dermatolog Treat 2022; 33:829–35. 32530334 10.1080/09546634.2020.1782322

[29] Kamel MM, Hegazy RA, Hegazy AA et al Combined subcision, autologous platelet-rich plasma, and CROSS technique in the treatment of atrophic acne scars: prospective split face study. Clin Dermatol 2021; 39:1018–24. 34920819 10.1016/j.clindermatol.2021.07.003

[30] Nassar SO, Eltatawy RAR, Hassan GFR. Safety and efficacy of platelet-rich plasma vs carboxytherapy in the treatment of atrophic scars: a comparative clinical and histopathological study. Dermatol Ther 2020; 33:e13942. 32608166 10.1111/dth.13942

[31] Gulanikar AD, Vidholkar R. Efficacy of platelet-rich plasma in acne scars. Clinical Dermatology Review 2019; 3:109–44.

[32] Zhang W, Guo Y, Kuss M et al Platelet-rich plasma for the treatment of tissue infection: preparation and clinical evaluation. Tissue Eng Part B Rev 2019; 25:225–36. 30712506 10.1089/ten.teb.2018.0309 PMC6589492

[33] Meretsky CR, Polychronis A, Schiuma AT. A comparative analysis of the advances in scar reduction: techniques, technologies, and efficacy in plastic surgery. Cureus 2024; 16:e66806. 39268283 10.7759/cureus.66806 PMC11392586

[34] Kim EY, Wong JH, Hussain A, Khachemoune A. Evidence-based management of cutaneous scarring in dermatology part 2: atrophic acne scarring. Arch Dermatol Res 2023; 316:19. 38059974 10.1007/s00403-023-02737-9

